# Interocular Symmetry in Anterior Segment Parameters in Children With Intermittent Exotropia Observed by CASIA2 AS‐OCT

**DOI:** 10.1155/joph/1051158

**Published:** 2026-09-18

**Authors:** Jiawei Zhao, Xiaoli Qi, Xiaoyu Zeng, Song Lin, Kadiya Abudukeyimu, Luning Qin, Zong Yu, Nan Wei, Gang Ding, Xue Li, Jing Li, Ning Hua, Xuehan Qian

**Affiliations:** ^1^ Tianjin Key Laboratory of Retinal Functions and Diseases, Tianjin Branch of National Clinical Research Center for Ocular Disease, Eye Institute and School of Optometry, Tianjin Medical University Eye Hospital, Tianjin, China, tmuec.com

## Abstract

**Purpose:**

To explore the interocular symmetry of the anterior segment parameters in subjects with intermittent exotropia (IXT) and compare it with that in the normal subjects.

**Methods:**

A total of 122 eyes of 61 subjects with IXT and 88 eyes of 44 normal subjects were enrolled. Central corneal thickness (CCT), the radii of curvature of the anterior and posterior lens (ALR and PLR), lens thickness (LT), lens equatorial diameter (DIA), anterior chamber depth (ACD), lens tilt, and decentration were measured with CASIA2 AS‐OCT; the parameters in IXT were measured again after cycloplegia. *T*‐tests, intraclass correlation coefficients (ICCs), and Bland–Altman plots were used to compare binocular differences and quantify symmetry. We compared the binocular symmetry between normal subjects and the subjects with IXT and the symmetry in different refractive states.

**Results:**

The ICCs were high in CCT, ACD, ALR, and LT and moderate in PLR, DIA, lens tilt, and decentration in all subjects (*p* < 0.05). IXT after cycloplegia showed similar ICCs to those of normal subjects, while ICCs before cycloplegia were all below normal. In the Bland–Altman plot, the IXT group had a larger mean difference and more dispersed 95% confidence intervals than the normal group. The normal group had similar ICCs for different refractive states, but hyperopic subjects had smaller ICCs than myopic subjects in the IXT group.

**Conclusions:**

IXT had lower binocular symmetry in the vision state than normal children, with the asymmetry being more pronounced in hyperopia. Binocular interocular asymmetry of children with IXT is of concern.

**Trial Registration:** Chinese Clinical Trial Registry: ChiCTR2300075756.

## 1. Introduction

Intermittent exotropia (IXT) is the most common type of strabismus, with a prevalence of about 1%–5% in China [1, 2]. The patients can only intermittently control positive positions; when visual fatigue or inattention occurs, binocular fusion is more likely to be broken, and the eye position tends to deviate outward when the deviated eye image is suppressed. Previous studies [3–5] have found that healthy patients were symmetrical in the structure and accommodative function of both eyes. In patients with IXT, there was significant accommodation asymmetry in both eyes; the dominant eye showed a higher accommodative response and accommodation sensitivity and lower accommodation lag compared to the nondominant eye [6, 7]. Given these findings and the important role of the crystalline lens in accommodation, we further investigated the interocular symmetry of the anterior segment and lens‐related biometric parameters in children with IXT.

Zhu et al. [8]found an association between myopia and exotropia. Previous population‐based observational studies [9, 10] suggested that children with IXT have a more pronounced tendency toward myopia. The 2022 Esotropia and Exotropia Preferred Practice Pattern [11] suggests that reducing and preventing the onset of myopia may reduce the incidence of strabismus. Some scholars argue that children with IXT need to use more accommodation and convergence to maintain binocular single vision, which may contribute to the development of myopia [12]. Assessing interocular symmetry in anterior segment parameters may improve our understanding of binocular characteristics in children with IXT and provide reference information for this population and establish a baseline for future studies investigating disease‐related structural changes or treatment outcomes.

The common instruments used to measure anterior segment parameters include ultrasound, which is a contact measurement that is difficult for children to cooperate with, and Pentacam, which is difficult to measure the posterior surface of the lens [13]. Choosing the appropriate instrument is important to improve the accuracy of measurements in children. CASIA2 AS‐OCT measures quickly and accurately [14]. Using it before and after cycloplegia can provide the anterior segment parameters under near vision conditions and lens relaxation conditions.

This study investigated the morphological characteristics of anterior segment structures in pediatric populations. The intraocular symmetry of anterior segment parameters was compared between normal children and children with IXT to evaluate the bilateral anterior segment morphology and determine whether structural symmetry is preserved in children with IXT.

## 2. Subjects and Methods

The study protocol was approved by the Tianjin Medical University Eye Hospital Review Board. All the procedures of this study followed the tenets of the Declaration of Helsinki, and informed consent was obtained from all the patients before enrollment. Children who visited the Tianjin Medical University Eye Hospital between November 2022 and July 2023 and met the inclusion and exclusion criteria were consecutively recruited into the study. A total of 61 children with IXT were included. Inclusion criteria were defined as follows: (1) patients with IXT can control positive positions in daily life, and deviation ≥ 15 prism diopters measured by the alternate prism cover test, with no difference between long and short distance; (2) relatively good binocular visual function (less than Grade 3 on Mohney’s and Holmes’ 6‐point control scale, corresponding to IXT present for less than 50% of the time during the 30‐s observation period, and near stereoacuity better than 400 s of arc on the Titmus stereo test, a threshold commonly used to indicate preserved functional stereopsis in children with IXT, indicating the ability to maintain functional binocular cooperation despite reduced fine stereoscopic resolution in children with IXT [15]; (3) low to moderate refractive error, anisometropia ≤ 0.50D, and astigmatism < 0.50D; and (4) the patients had no experience using atropine and orthokeratology lenses that could affect the structure of the anterior segment before measurement. Exclusion criteria were defined as follows: amblyopia, nystagmus, A and V patterns, vertical strabismus, dissociated vertical strabismus, paralytic or restrictive exotropia, ocular disease other than strabismus and refractive error, and recently received accommodative training. We also recruited 44 normal children. These children were required to have anisometropia ≤ 0.50D, astigmatism < 0.50D, and no eye disease other than refractive error. Spherical equivalent (SE) was calculated by the formula SE = spherical + cylindrical/2. Myopia was defined as an SE < −0.50 *D*; hyperopia was defined as an SE > +0.50 *D*.

In this study, CASIA2 AS‐OCT (Tomey Corporation, Nagoya, Japan) was used to measure the anterior segment parameters. This instrument can image the anterior portion of the eye using a 1310‐nm wavelength at a rate of 50,000 A‐scans per second, with a measurement depth of approximately 13 mm and a width of approximately 16 mm. With the Pre‐op Cataract mode, 16 radial 2D tomographic images can be acquired at one time, and the 3D parameters of the lens are automatically calculated by the built‐in software of CASIA2 [16, 17]. The CASIA2 AS‐OCT has good repeatability and accuracy [14, 18].

Central corneal thickness (CCT), the radii of curvature of the anterior lens (ALR), the radii of curvature of the posterior lens (PLR), lens thickness (LT), lens equatorial diameter (DIA), anterior chamber depth (ACD), lens tilt, and lens decentration were measured using CASIA2 AS‐OCT in both eyes of IXT and normal children in the visual state. All examinations were performed by a trained operator under the same room lighting conditions and in a standardized examination setting. Subjects were instructed to maintain fixation on the internal target during measurements. To ensure that measurements in the IXT group were obtained under the visual condition rather than the manifest exotropia state, a second examiner continuously monitored ocular alignment throughout the examination.

In patients with IXT, cycloplegia was induced using compound tropicamide eye drops (Shenyang Xingqi, China), administered four times at 5‐min intervals. Postcycloplegic measurements were performed 20 min after the final instillation. All measurements were performed at the same location under identical lighting conditions.

## 3. Statistical Analysis

Statistical analysis was performed using SPSS software (Version 20.0). Bland–Altman plots were generated using MedCalc (Version 23.0.2). Because the primary objective of this study was to evaluate interocular symmetry, all analyses were performed using paired data from both eyes of the same subject, taking into account within‐subject correlation. All parameters were confirmed using the Kolmogorov–Smirnov test for the normality of the data distribution and were expressed using mean ± standard deviation including non‐normally distributed data. Paired *t*‐tests and Wilcoxon signed‐rank tests were used to compare the differences between both eyes. Interocular correlations were assessed using the Spearman correlation coefficient. Intraclass correlation coefficients (ICCs) were calculated to evaluate the degree of agreement between both eyes. ICC values < 0.50 were considered poor, 0.50–0.75 moderate, 0.75–0.90 good, and > 0.90 excellent agreement. Correlation coefficients (*r*) > 0.80 were considered to indicate strong correlation. A *p* value < 0.05 was considered statistically significant.

## 4. Results

A total of 122 eyes of 61 subjects with IXT and 88 eyes of 44 normal subjects were included. The IXT group included 30 myopic and 31 hyperopic subjects, while the normal group included 19 myopic, 22 hyperopic, and three emmetropic subjects. Table 1 shows the demographic data of all the subjects.

**TABLE 1:** Patient demographics and clinical characteristics.

	IXT group	Normal group	*p*
*n*	61	44	—
Sex (F/M)	34:27	22:22	0.561
Age (y)	7.85 ± 1.82	8.34 ± 2.10	0.082
SE (D)			
Deviating eyes	−0.40 ± 1.40 (−2.01, +2.01)	—	0.833
Fixating eyes	−0.42 ± 1.43 (−2.06, +2.32)	—	
Right eyes	—	0.62 ± 1.43 (−1.38 + 1.63)	0.943
Left eyes	—	0.50 ± 1.34 (−1.50, +1.50)	

*Note:* SE, spherical equivalent; SE was expressed using mean ± standard (Q1, Q3).

There was no significant difference in the anterior segment parameters between both eyes in the subjects with IXT and the normal subjects (*p* > 0.05). Table 2 shows the difference in the anterior segment parameters in both eyes in the IXT (precycloplegia) and normal groups.

**TABLE 2:** Anterior segment parameters in both eyes in the IXT (precycloplegia) and normal groups.

	IXT group (precycloplegia)			Normal group		
	Deviating eyes	Fixating eyes	*p*	Right eyes	Left eyes	*p*
ALR (mm)	12.00 ± 1.55	11.95 ± 1.64	0.651	11.82 ± 1.98	11.76 ± 1.73	0.100
PLR (mm)	5.88 ± 0.44	5.79 ± 0.36	0.156	5.85 ± 0.48	5.83 ± 0.50	0.876
LT (mm)	3.52 ± 0.16	3.52 ± 0.16	0.622	3.50 ± 0.22	3.51 ± 0.23	0.196
DIA (mm)	9.53 ± 0.35	9.47 ± 0.27	0.101	9.42 ± 0.37	9.42 ± 0.37	0.933
CCT (μm)	534.08 ± 33.47	534.41 ± 31.59	0.841	534.67 ± 33.82	535.83 ± 34.15	0.055
ACD (mm)	3.29 ± 0.28	3.30 ± 0.28	0.884	3.20 ± 0.31	3.18 ± 0.35	0.413
Tilt (°)	4.71 ± 1.11	4.72 ± 1.11	0.947	4.93 ± 1.22	5.23 ± 1.19	0.089
Decentration(mm)	0.17 ± 0.09	0.18 ± 0.07	0.156	0.17 ± 0.08	0.16 ± 0.06	0.270

Abbreviations: ACD, anterior chamber depth; ALR, radius of lens anterior surface; CCT, central corneal thickness; DIA, equivalent diameter of the lens; LT, crystalline lens thickness; PLR, radius of lens posterior surface.

**FIGURE 1:**
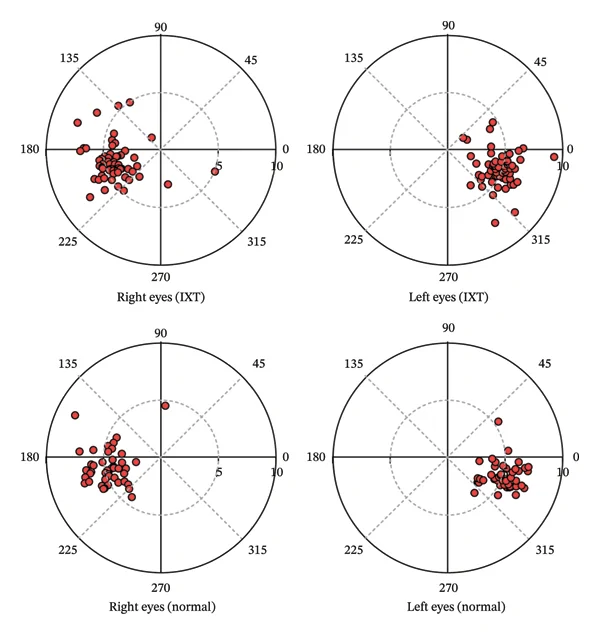
Distribution of crystalline lens decentration in IXT and normal subjects. Each point represents the decentration direction of one eye relative to the corneal vertex. The directional angles corresponding to lens decentration were 0° on the left side, 90° on the superior side, 180° on the right side, and 270° on the inferior side.

**FIGURE 2:**
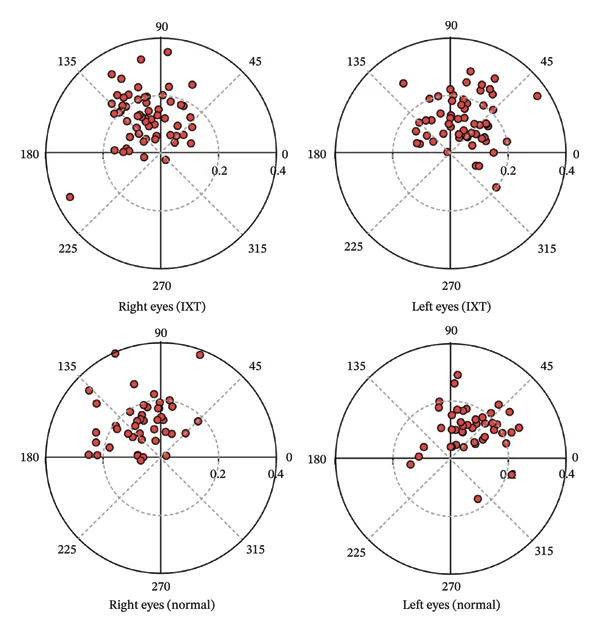
Distribution of crystalline lens tilt in IXT and normal subjects. Each point represents the tilt direction of one eye relative to the corneal vertex. The directional angles corresponding to lens tilt were 0° on the left side, 90° on the superior side, 180° on the right side, and 270° on the inferior side.

The crystalline lens tilted mainly toward the inferotemporal direction. The crystalline lens decentered toward the superior side in the IXT group and toward the superior temporal side in the normal group (Figures 1 and 2).

In both normal children and children with IXT, binocular ALR, LT, CCT, and ACD are highly correlated, and binocular DIA, PLR, lens tilt, and decentration are moderately correlated before and after cycloplegia (Table 3).

**TABLE 3:** The correlation of anterior segment parameters in both eyes.

	Normal		IXT (postcycloplegia)		IXT (precycloplegia)	
	r	*p*	r	*p*	r	*p*
ALR (mm)	0.923	**< 0.001**	0.966	**< 0.001**	0.858	**< 0.001**
PLR (mm)	0.720	**< 0.001**	0.634	**< 0.001**	0.389	**0.002**
LT (mm)	0.946	**< 0.001**	0.972	**< 0.001**	0.938	**< 0.001**
DIA (mm)	0.739	**< 0.001**	0.706	**< 0.001**	0.466	**< 0.001**
CCT (μm)	0.986	**< 0.001**	0.991	**< 0.001**	0.963	**< 0.001**
ACD (mm)	0.968	**< 0.001**	0.982	**< 0.001**	0.862	**< 0.001**
Tilt (°)	0.580	**< 0.001**	0.456	**0.017**	0.450	**< 0.001**
Decentration (mm)	0.677	**< 0.001**	0.580	**0.002**	0.416	**< 0.001**

*Note:* The bold values indicate *p* < 0.05.

Abbreviations: ACD, anterior chamber depth; ALR, radius of lens anterior surface; CCT, central corneal thickness; DIA, equivalent diameter of the lens; LT, crystalline lens thickness; PLR, radius of lens posterior surface.

**FIGURE 3:**
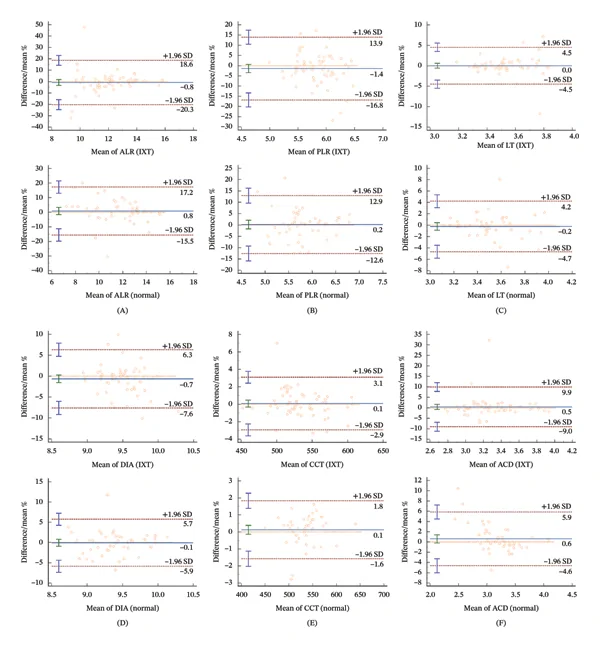
Bland–Altman plots of the anterior segment parameters in both eyes in the IXT (precycloplegia) and normal groups. The solid line represents the mean interocular difference, and the dashed lines indicate the 95% limits of agreement (mean ± 1.96 SD). Narrower limits of agreement indicate better interocular consistency. (A) (B) (C) (D) (E) (F).

The ICCs were high in binocular CCT, ACD, ALR, and LT and moderate in PLR, DIA, lens tilt, and decentration in all subjects. Subjects with IXT after cycloplegia had similar ICCs to those of normal subjects, and the ICCs were higher than those in the IXT group before cycloplegia (Table 4). Figure 3 demonstrates the Bland–Altman plots of the anterior segment parameters in both eyes in the IXT (precycloplegia) and normal groups. The IXT group had a larger mean difference and more dispersed 95% confidence intervals than the normal group.

**TABLE 4:** The ICCs of anterior segment parameters in both eyes.

	Normal		IXT (postcycloplegia)		IXT (precycloplegia)	
	ICC	*p*	ICC	*p*	ICC	*p*
ALR (mm)	0.923	**< 0.001**	0.966	**< 0.001**	0.795	**< 0.001**
PLR (mm)	0.736	**< 0.001**	0.564	**< 0.001**	0.317	**0.006**
LT (mm)	0.938	**< 0.001**	0.983	**< 0.001**	0.863	**< 0.001**
DIA (mm)	0.727	**< 0.001**	0.654	**< 0.001**	0.421	**< 0.001**
CCT (μm)	0.995	**< 0.001**	0.991	**< 0.001**	0.969	**< 0.001**
ACD (mm)	0.974	**< 0.001**	0.989	**< 0.001**	0.847	**< 0.001**
Tilt (°)	0.553	**< 0.001**	0.484	**0.049**	0.46	**< 0.001**
Decentration (mm)	0.639	**< 0.001**	0.546	**< 0.001**	0.402	**0.001**

*Note:* The bold values indicate *p* < 0.05.

Abbreviations: ACD, anterior chamber depth; ALR, radius of lens anterior surface; CCT, central corneal thickness; DIA, equivalent diameter of the lens; LT, crystalline lens thickness; PLR, radius of lens posterior surface.

We calculated the ICCs for myopic and hyperopic subjects in the two groups in the precycloplegia state. The ICCs for the different refractive states were similar in the normal group, but in the IXT group, the ICCs were lower in hyperopic subjects than in myopic subjects. Table 5 demonstrates the ICCs of anterior segment parameters in different refractive states.

**TABLE 5:** The ICCs of anterior segment parameters in different refractive states.

	IXT group				Normal group			
	Myopia		Hyperopia		Myopia		Hyperopia	
	ICC	*p*	ICC	*p*	ICC	*p*	ICC	*p*
ALR (mm)	0.901	**< 0.001**	0.614	**< 0.001**	0.905	**< 0.001**	0.972	**< 0.001**
PLR (mm)	0.367	**0.021**	0.237	0.096	0.714	**0.010**	0.849	0.096
LT (mm)	0.941	**< 0.001**	0.795	**< 0.001**	0.955	**< 0.001**	0.971	**< 0.001**
DIA (mm)	0.515	**0.002**	0.303	**0.046**	0.828	**0.002**	0.833	**0.046**
CCT (μm)	0.976	**< 0.001**	0.965	**< 0.001**	0.993	**< 0.001**	0.990	**< 0.001**
ACD (mm)	0.980	**< 0.001**	0.754	**< 0.001**	0.980	**< 0.001**	0.981	**< 0.001**

*Note:* The bold values indicate *p* < 0.05.

Abbreviations: ACD, anterior chamber depth; ALR, radius of lens anterior surface; CCT, central corneal thickness; DIA, equivalent diameter of the lens; LT, crystalline lens thickness; PLR, radius of lens posterior surface.

## 5. Discussion

In this study, binocular symmetry was reduced in subjects with IXT before cycloplegia but improved after cycloplegia to a level comparable with that of noncycloplegic normal controls. Within the IXT group, hyperopic subjects exhibited lower binocular symmetry than myopic subjects. We also found that in children, lens decentration toward superior temporal positions differed from inferotemporal or temporal regions reported in previous studies.

Previous studies have found that the symmetry was high in ocular parameters in normal adults [19], but there are no relevant studies in children with IXT. The anterior segment parameters before cycloplegia reflect the parameters in the near vision state, and the parameters after cycloplegia are considered the parameters under relaxation of accommodation [20]. In this study, we found that in subjects with IXT, there were no significant differences in anterior segment parameters between the deviating and fixating eyes, and the ICCs after cycloplegia were similar to those of the normal subjects. These findings suggest that IXT is not associated with intrinsic bilateral structural abnormalities of the anterior segment. However, a modest reduction in interocular consistency was observed under visual conditions, indicating that functional factors related to accommodation may influence biometric symmetry in IXT. These findings provide a useful reference for interpreting anterior segment biometric measurements in children with IXT and suggest that accommodative state should be considered when evaluating interocular consistency. The relatively lower ICC values observed for PLR and lens decentration may reflect greater susceptibility of these parameters to accommodative influences under visual conditions, although further studies are needed to confirm this interpretation. It should be noted, however, that normal subjects in this study were examined only under noncycloplegic conditions. It remains unclear whether cycloplegia would further improve the binocular symmetry of anterior segment parameters in normal eyes. Future studies incorporating cycloplegic measurements in normal subjects are required to establish normative symmetry values under cycloplegic conditions and to more precisely define the extent of symmetry recovery in IXT.

There was no significant difference between the accommodation of the two eyes in normal subjects [21], and when one eye accommodates, the other eye is linked to produce an equal amount of accommodation. Yang [6] has found that patients with IXT with a marked deviating eye tend to have asymmetric accommodative responses between both eyes; the deviating eye shows a more obvious accommodation lag. Chow A [22] found differences in the accuracy of binoculars in patients with strabismic amblyopia, whereby attention was biased toward the dominant eye. Kwon [23] found that patients with IXT had significantly lower binocular contrast sensitivity than healthy subjects. However, influenced by the instrumentation and the child’s cooperation, previous studies have rarely explored intraocular structures in children with IXT. We found that although subjects with IXT (precycloplegia) had high binocular symmetry (ICCs > 0.75), the overall ICCs of the anterior segment were lower than in those in normal children. This may be due to asymmetric accommodation in strabismic subjects to ensure a positive position or the presence of monocular suppression that causes accommodation relaxation in deviating eyes. The observed improvement in interocular symmetry after cycloplegia suggests that accommodative activity plays a role in the modest reduction of biometric consistency seen under visual conditions in children with IXT. Gong [24] also found that the symmetry of the anterior segment parameters was lower in subjects with IXT than in normal patients, but they only measured binocular ICC values for the ACD and LT, which were 0.955 and 0.924 respectively, higher than those in our study. Overall, these findings suggest that accommodative imbalance under visual conditions, rather than structural asymmetry, may be a key factor contributing to the reduced interocular consistency observed in children with IXT.

In the present study, we found that the binocular symmetry in normal children was not significantly different in different refractive states, but the hyperopic subjects exhibited lower binocular symmetry than myopic subjects in the IXT group. This finding suggests that refractive status may be associated with differences in binocular symmetry in children with IXT. We know that patients with IXT can maintain a positive position through binocular accommodation and convergence [25], and refractive status may influence this control strategy. One potential explanation is that hyperopic subjects may rely more on accommodative effort to maintain a positive position, which could introduce greater asymmetry in anterior segment parameters.

However, direct evidence supporting increased accommodative compensation specifically in hyperopic IXT patients is currently limited, and this interpretation should be regarded as speculative. Alternatively, the observed difference in symmetry may be related to other factors, such as the relatively small sample size in refractive subgroups or differences in age distribution between hyperopic and myopic subjects. It should be noted that the subgroup analysis by refractive status (myopia vs. hyperopia) was performed on a limited sample size; therefore, these results should be considered exploratory and need to be confirmed in future studies with larger cohorts.

Most previous studies have found lens decentration toward the inferotemporal or temporal regions [26, 27]. In contrast, we observed lens decentration toward the superior temporal positions in normal children and toward the superior positions in children with IXT. The direction of lens tilt and decentration did not differ significantly between both eyes. The children included in this study were 6–12 years old, and we know that lens density increases with age [28]; thus, lens decentration in this study is more upward than in previous studies, which may account for the lower lens density [29, 30]. These observations may provide useful reference data for future studies investigating lens morphology and anterior segment biometrics in pediatric strabismus.

Some limitations remain in our study. First, this was a cross‐sectional study with a relatively limited sample size, and longitudinal changes in anterior segment symmetry could not be evaluated. Therefore, the dynamic relationship between accommodative status and interocular symmetry over time remains unclear. In addition, AS‐OCT CASIA2 can only measure parameters during monocular viewing. Therefore, the results obtained in this study may not fully represent binocular viewing conditions in patients with IXT in daily life. Second, we could not obtain adequate anterior segment measurements after cycloplegia in normal subjects. Therefore, we only compared postcycloplegic interocular symmetry in children with IXT to previously reported normal data. The included patients had relatively good binocular function; thus, future studies are needed to investigate whether patients with constant strabismus exhibit more pronounced interocular asymmetry than those with IXT.

In this study, we found that children with IXT showed generally symmetrical anterior segment structures between both eyes but exhibited reduced interocular symmetry compared with normal children under viewing conditions, with greater asymmetry in hyperopic subjects. These findings provide insight into the anterior segment characteristics of IXT.

## Funding

The study was funded by the Tianjin Key Medical Discipline Construction Project (TJYXZDXK‐3‐004A‐2).

## Conflicts of Interest

The authors declare no conflicts of interest.

## Data Availability

The data that support the findings of this study are available from the corresponding author upon reasonable request.
